# Overview of Checkpoint Inhibitors Mechanism of Action: Role of Immune-Related Adverse Events and Their Treatment on Progression of Underlying Cancer

**DOI:** 10.3389/fmed.2022.875974

**Published:** 2022-05-30

**Authors:** Patricia Iranzo, Ana Callejo, Juan David Assaf, Gaspar Molina, Daniel Esteban Lopez, David Garcia-Illescas, Nuria Pardo, Alejandro Navarro, Alex Martinez-Marti, Susana Cedres, Caterina Carbonell, Joan Frigola, Ramon Amat, Enriqueta Felip

**Affiliations:** ^1^Medical Oncology Department, Vall d'Hebron University Hospital, Vall d'Hebron Institute of Oncology, Barcelona, Spain; ^2^Thoracic Cancers Translational Genomics Unit, Vall d'Hebron Institute of Oncology (VHIO), Barcelona, Spain

**Keywords:** immune checkpoint inhibitors (ICIs), immune-related adverse events (irAEs), corticosteroids, efficacy, multidisciplinary management

## Abstract

In recent years, immunotherapy-based regimens have been included into the treatment's algorithm of several cancer types. Programmed death-1 (PD-1) and cytotoxic T lymphocyte antigen-4 (CTLA-4) interact with their ligands found on the surface of antigen presenting cells (APC) or tumor cells (PD-L1/2 and CD80/86). Through these interactions, stimulatory or inhibitory signals are established. Immune checkpoint inhibitors (ICIs), block these interactions, and when administered not only as monotherapy but also as part of combination regimens, have shown to improve survival results in multiple advanced cancers leading to an increasing number of patients treated with ICI and, as a consequence, a rise in the number of patients developing immune-related adverse events (irAEs). Presence of irAEs has been associated with greater benefit from treatment, especially when blocking PD-L1. Recent data suggests that treatment benefit persists after discontinuation of ICIs due to a treatment related adverse event, regardless of the grade. Patients experiencing grade 3-4 irAEs are at risk of toxicity recurrence after reintroducing immunotherapy and therefore, the decision to resume the treatment is challenging. In these cases, a multidisciplinary approach is always needed and several factors should be considered. Management of severe toxicities may require systemic corticosteroids which can impact on T-cell function. Due to their immunosuppressive properties, it is necessary to deeper determine how corticosteroids influence responses. In terms of overall survival (OS), the use of steroids as therapy for irAEs seems not to reduce OS and several studies have reported durable responses in patients experiencing autoimmune toxicities treated with corticosteroids.

## Introduction

Over the last decade, immunotherapy has radically changed cancer therapy.

Since 2011, when Food and Drug Administration (FDA) approved ipilimumab to treat patients with late-stage (metastatic) melanoma (1), several immunotherapies have received regulatory authorities' approval. Different cancer types have shown remarkable responses to this therapy (2, 3). ICIs, as monotherapy but also as part of a combination therapy, improve results in terms of Progression Free Survival (PFS) and Overall Survival (OS) (4–7).

The first tumor types in which immunotherapy was introduced as part of their treatment algorithms were melanoma, Renal Cell Carcinoma (RCC) and Non-Small-Cell Lung Cancer (NSCLC).

Drugs targeting two different checkpoint axis, based on the T-cell membrane (PD-1 and CTLA-4), have shown clinical activity and, indeed, concentrate the biggest evidence in terms of disease control and the largest number of drugs approved and introduced into the clinical practice. Through their interaction with ligands found on the surface of antigen presenting cells (APC) or tumor cells, the immune response is modulated.

Immunotherapy is presented with a specific toxicity profile with diverse types of inflammatory-mediated side effects. The incidence and characteristics of the different adverse events associated with ICIs depend on the patients' profile, cancer diagnosis and type of agent used.

The most common toxicities of ICIs occur at the skin, gastrointestinal mucosa, liver, endocrine glands and respiratory tract (8) but almost every tissue or organ can be affected.

From the pathophysiological point of view, both benefit and toxicity occur as consequence of immune activation. Due to this common etiology, an association between the appearance of irAEs and the benefit of immunotherapy has been proposed (9).

Thus, the development of irAEs has been suggested to be predictive of improved tumor response and better survival in some cancer patients treated with ICIs. However, the occurrence of irAE is not strictly necessary for achieving treatment's benefit (10).

Nowadays, an increasing number of co-stimulatory and co-inhibitory signals participating in the immune response are being identified and targeted. The knowledge about the interactions between them focuses most of the research on this field.

These advances, in terms of disease control and survival, have led to a very significant increase in the number of patients treated with immunotherapy. This large volume of patients receiving immunotherapy has highlighted the need to improve the understanding of the mechanisms of action, the interrelation between the immune signals and the potential toxicity profiles.

Therefore, to improve patient selection it is necessary to consider predictive biomarkers of benefit but also to ensure a correct assessment of their susceptibility to develop irAEs.

## Mechanisms of Action of ICIs

The immune system protects against tumor growth but also modifies tumor immunogenicity (11). During this process, some tumor cells escape to the antitumor immune response using different mechanisms involving antigens, cytokines and immune checkpoint proteins (12).

Understanding tumor immunology must be achieved through the integration of local immune response in the tumor microenvironment with the changes in the peripheral immune system (13). Immunity in cancer is regulated by diverse cell types in different tissues so its activation or inhibition through cancer immunotherapies may lead to immune responses potentially involving different organs.

Monoclonal antibodies that block the regulatory immune targets CTLA-4, PD-1 and programmed death-1 ligand (PD-L1) are the most well-studied and have the biggest evidence as cancer immunotherapies.

CTLA-4 is present on the surface of CD4-positive and CD8-positive lymphocytes and binds to T-cell–costimulatory factors on the surface of APC. CTLA-4 binding reduces interleukin 2 (IL-2) production and T-cell proliferation.

PD-1 is a receptor expressed on the surface of multiple immune cell types, including T cells, B cells, and NK cells. One of its ligands, PD-L1, is present in different cell types including tumor cells and participates in the inhibition of previously activated T cells.

Approved ICIs include anti-PD1 antibodies (pembrolizumab, nivolumab, cemiplimab), anti-PD-L1 (atezolizumab, avelumab, and durvalumab) and anti–CTLA-4 (ipilimumab, tremelimumab).

In the last few years, a deeper understanding of tumor immunology has led to an increasing number of immunotherapies in clinical development (e.g., blockade of LAG3, TIGIT and TIM3) (14).

The aforementioned pathways can be used by tumor cells to evade the immune system mainly through the inhibition of T-cell function (15).

Checkpoint blockade using ICIs overcomes this tumor-mediated immune inhibition, leading to a proinflammatory tumor microenvironment which potentially increases the disease control but also the risk of triggering an inflammatory-mediated toxicity. ICIs response and toxicity are closely related because of the disinhibition of T-cell3 function. Notably, even in with no history of autoimmune disorders prior to initiation of treatment, irAEs may appear.

T-cells infiltrate is considered to be responsible for both the anti-tumor response and the development of immune-toxicities but, beyond T-cells, a much more complex inflammatory interaction occurs within the immune response.

## Mechanisms of irAEs

The pathophysiology of irAEs is still under investigation and is not fully understood. Several mechanisms are hypothesized as possible contributors in the development of immune-mediated effects. Autoantibodies, T-cell infiltration, interleukins and other inflammatory cytokines have been proposed to account for the occurrence of irAEs (16).

Regarding autoantibodies, in a study of patients treated with ICIs, the identification of autoantibodies correlated with the development of hypophysitis and pneumonitis (17). Another study of patients treated with pembrolizumab showed that, up to 80% of patients who developed hypothyroidism had antithyroid antibodies compared with 8% of patients with normal thyroid function (18).

Cytokines levels, at baseline but also after the treatment, have been associated with the development of irAEs (19).

CTLA-4 related adverse events are different from those developed with anti-PD1 therapy since CTLA-4 inhibits T cells in the beginning of the immune response while PD-1 blocks T-cell in peripheral tissues and in a more advanced step of the immune response.

The interaction or relationship between benefit and toxicity, in terms of immune related effects, has been reported in different studies (20, 21) and a deeper knowledge of this interplay will facilitate the identification of risk factors and will help to implement prevention and follow-up strategies.

## Association Between irAEs and Prognosis in Solid Tumors

Immunotherapeutic agents are widely used in different types of advanced tumors as melanoma, lung cancer, renal clear cell cancer, head and neck cancer and gastrointestinal cancers among others.

There is a subset of patients who benefit most from immunotherapy with long-term survival. The identification of these patients through biomarkers or specific features has been a crucial point for the scientific community in recent past years (22).

In retrospective studies, the presence of irAEs has been associated with clinical benefit. ICIs can induce side effects through the inflammation with lymphocyte infiltration at any organ and consequently a system dysfunction. Most irAEs are mild and transient, nevertheless, sometimes they can be life-threatening. In fact, this can limit retreatment with ICIs after a toxicity or also it can lead to permanent dysfunctions and in some cases, patients may not recover from the adverse event. IrAEs not only affect the immunotherapy rechallenge, they may also impact in the potential subsequent antineoplastic treatment that the patient will receive, especially if the patient does not recover the adequate organ function, and finally, they can impact on patients survival.

Despite this, recent publications have reported a relationship between irAEs and clinical efficacy in cancer patients in terms of response rate, PFS and OS (23).

In the case of lung cancer, a comprehensive retrospective study trying to identify biomarkers of long-term responders in advanced NSCLC patients that received ICI, suggests the presence of irAEs as a prognostic factor for better survival (24). In the same line, another publication of NSCLC patients treated with nivolumab in advance setting, has shown that the development of irAEs is associated with better PFS [9.2 months(m) vs. 4.8 m; HR = 0.52] and OS (NR vs. 11.1 m; HR = 0.28) (25). Similarly, positive association between irAEs and survival outcome has been demonstrated in a large cohort of NSCLC Italian patients treated with anti-PD1 agents. Specifically, higher ORR, longer PFS and longer OS were observed in patients who developed irAEs compared to those who did not. Of note, the median OS (mOS) in patients with irAEs was 20.50 vs. 8.5 m, irrespective of the type of irAE (26). In a retrospective French cohort of 270 patients the outcomes were also better in patients with irAEs, showing an OS NR vs. 8.21 m, respectively (HR = 0.2); the PFS was 5.2 vs. 1.97 m (HR = 0.42); and ORR was 21.3 vs. 5.7% (27). Similar data has been observed in an Asian study about patients treated with ICIs in which DFS is higher in the subset of patients who developed toxicity (28). Other similar series have been published reporting similar outcomes (29, 30).

Moreover, in NSCLC setting the influence of multisystem irAEs in survival has been researched and the presence of an irAE in more than one system or organ is associated with improved survival (21).

Also in melanoma cancer patients, a relationship has been described between irAEs and clinical outcomes. Longer mOS has been reported in melanoma patients treated with ICIs who presented toxicity compared to those who did not (21.9 vs. 9.7 m), respectively (31). Higher disease control rate has also been reported in patients with irAEs (69.8 vs. 49.3%) (32). In a real-world cohort including almost 200 patients, a greater OS and PFS was observed in melanoma patients who experienced irAEs than in those who did not, with reported data of NR vs. 9 m and 28 m vs. 5 m, respectively (33).

Focusing on the severity of the toxicity, a Canadian cohort of advanced melanoma patients treated with anti-PD1 agents observed a mOS of 39 vs. 23 m for any irAE and no irAE, respectively, and mOS NR vs. 29 m for grade ≥ 3 irAEs and no grade ≥ 3 irAEs, respectively (34).

Despite this data, some studies have reported controversial results regarding the association between irAEs and efficacy with ICI, showing no statistically significant better outcomes in patients with toxicity (35) and similar ORR (58.3 vs. 50.2%) (36).

In other solid tumors, this interaction between toxicity and results, has been confirmed. A retrospective study which included renal cell cancer patients demonstrated better PFS in patients with irAEs, although this benefit was not reflected in OS (37). In a study in renal cell cancer patients treated with anti-PD1 agents, a greater OS was reported for patients experiencing toxicity vs. those without toxicity (35.9 vs. 26.5 m, respectively) (38).

In head and neck cancer and gastrointestinal tumors, better outcomes have been reported too in those patients with irAEs vs. those without toxicity (39, 40).

Lastly, a meta-analysis which includes most relevant studies of different types of tumors has demonstrated a positive association between irAEs and survival regardless of the localization of the primary tumor, type of ICI and irAE (41).

Table 1 summarizes the results about the impact of irAEs and corticosteroids in terms of PFS and OS in the different types of tumors.

**Table 1:** Type of tumor and OS, PFS of different studies.

**Type of tumor**	**References**	**OS (months)**	**PFS (months)**		
		**irAEs**	**Without irAEs**	**irAEs**	**Without irAEs**
NSCLC	Haratani et al. (25)	NR	11.1	9.2	4.8
	Cortellini et al. (26)	20.5	8.5	10.1	4.1
	Grangeon et al. (27)	NR	8.2	5.2	2
	Ahn (42)	24	11.6	7.4	3.3
	Ricciuti et al. (29)	17.8	4	8.5	2
Melanoma	Indini et al. (31)	21.9	9.7	NA	NA
	Bastacky et al. (33)	NR	9	28	5
	Sou et al. (34)	39	23	NA	NA
Renal cell	Labadie et al. (37)	NA	NA	20.5	10.1
	Elias et al. (38)	35.9	26.5	17.8	6.6
Head and neck	Foster et al. (39)	12.5	6.8	6.9	2.1
Gastrointestinal	Das et al. (40)	32.4	8.5	32.4	4.8

## Impact of Steroids, Immunosuppressive Treatment and Antibiotics in Clinical Outcomes in Patients Who Develop irAEs

Corticosteroids are the mainstay in the management of toxicities produced by immunotherapy but in some cases the management of the toxicity does not require their use. It is known that the use of corticosteroids produces immunosuppression that could lead to tumor progression. However, whether the patients who needs steroids to manage the irAE have different prognosis compared to those who do not remains an unanswered question.

In order to investigate this point, a metanalysis was recently published suggesting a worse OS in patients taking steroids for supportive care reasons, but if the purpose of the treatment is to manage adverse events related with immunotherapy the OS was not affected (43).

These data are consistent with another study including different types of tumors. They observed that patients with irAEs that required steroids presented higher PFS but no differences in OS (44).

Following the same line, patient survival has not been affected by the use or not of immunosuppressants in the context of toxicity due to immunotherapy in patients with melanoma (45).

In conclusion, the published data suggest that the use of steroids to manage irAEs does not impact in the survival of the patients.

Antibiotics may also be potentially useful in treating irAEs. Antibiotics therapy led to an antibiotic-associated dysbiosis that appears to be detrimental to ICI efficacy (46). Several studies have evaluated this situation, but the evidence on the impact of antibiotics used to treat an irAE on the benefit of immunotherapy is much more limited (47). In a recent systematic review and meta-analysis, OS and PFS in patients treated with immunotherpy were negatively associated with the use of antibiotics but varies significantly between different types of tumors (48).

However, these conclusions about the impact of corticosteroids and antibiotics on ICIs benefit must be interpreted with caution due to the retrospective design and the low level of evidence of the majority of the studies published on these topics.

## Discussion

Diagnosis and management of irAEs is challenging and requires continuously updated diagnostic and monitoring tools.

Given that different immune checkpoint inhibitors may have distinct mechanisms of action, the incidence, severity and the tissue affected may vary.

The incidence of irAEs upon ipilimumab treatment (anti-CTLA4) is dose dependent, with up to 80% of patients experiencing some adverse events when treated at a dose of 10 mg/kg (49). Rates of irAEs with anti-PD-1/PD-L1 treatment are similar to those anti-CTLA4 and range from 70 to 85% but severe toxicities (G3-4) are less frequent (50).

Several factors can impact on ICIs treatment outcome. irAEs and their treatment are one of the most studied.

This is especially important given that the immune mechanisms involved in disease control are, in many cases, very similar to those that trigger immune-mediated toxicities. Therefore, treating the secondary effects can generate a decrease in immune activity and, as a consequence, a lower efficacy of the treatment (51).

The development of an adverse effect may have multiple consequences. The inflammation of the organ or tissue can be permanent and lead to organ failure. In addition, toxicity may be associated with clinical deterioration of the patient. All of this can limit or condition the use of subsequent treatments and impact the patient's survival and quality of life. However, with irAEs, this negative impact of permanent sequelae, is under debate and is conditioned by different factors and clinical situations.

Corticosteroids and antibiotics are the most commonly prescribed medications for the treatment of AEs during immunotherapy and both of them can impact on ICIs treatment efficacy.

Due to their immunosuppressive effects, treatment with corticosteroids is associated with worse outcomes in terms of efficacy (52). However, the time at which they are initiated and the reason for which they are prescribed seem to play a role in the consequences of their use on the disease control.

When administered to control the symptoms of the disease, they have a negative effect on the efficacy that does not seem to be equally obvious when they are used in the context of an irAE (53).

Further research is needed to improve the knowledge about the interactions created between the different checkpoints involved in the immune response. Due to this increasing complexity, a multidisciplinary team is necessary to ensure an optimal management of these toxicities that can become serious and/or permanent.

## Author Contributions

All authors listed have made a substantial, direct, and intellectual contribution to the work and approved it for publication.

## Conflict of Interest

AC reports advisory role and/or travel compensation: Bristol-Myers Squibb Recipient, F. Hoffmann La Roche AG, Pfizer, Boehringer Ingelheim, MSD Oncology, Leo Pharma, Medscape, and Kern Pharma. PI reports advisory role and/or travel compensation: Bristol-Myers Squibb Recipient, F. Hoffmann, La Roche AG, Merck Sharp & Dohme, Boehringer Ingelheim, MSD Oncology, Rovi, Kyowa Kirin, Grunenthal Pharma S.A., Pfizer, Medscape, Kern Pharma. NP reports advisory role and/or travel compensation: MSD oncology, Merck Sharp & Dohme, Bristol-Myers Squibb Recipient, F. Hoffmann La Roche AG, Pfizer, Boehringer Ingelheim, Grunenthal Pharma S.A Kern Pharma. AN reports advisory role, speaker's bureau or travel compensation: Bristol-Myers Squibb, F. Hoffmann La Roche AG, Pfizer, Boehringer Ingelheim, Oryzon Ge-nomics, Pfizer, AstraZeneca. AM-M provided consultation, attended advisory boards and/or speaker's bureau for the following organizations: Bristol-Myers Squibb, Lilly, F. Roche, MSD oncology, Pfizer, Boehringer Ingelheim, AstraZeneca. SC Bristol-Myers Squibb Recipient F, Hoffmann La Roche AG, Pfizer, Boehringer Ingelheim, MSD Oncology, Amphera. CC and JF had been partially supported by Grant for Oncology EMD Serono research funding to EF. EF reports consulting, advisory role or speaker's bureau: AbbVie, AstraZeneca, Blueprint medicines, Boehringer Ingelheim, Bristol-Myers Squibb, Celgene, Eli Lilly, Guardant Health, Janssen, Medscape, Merck KGaA, Merck Sharp & Dohme, Novartis, Pfizer, Roche, Takeda, Touchtime and research funding with: Fundación Merck Salud, Grant for Oncology Innovation EMD Serono. The remaining authors declare that the research was conducted in the absence of any commercial or financial relationships that could be construed as a potential conflict of interest.

## Publisher's Note

All claims expressed in this article are solely those of the authors and do not necessarily represent those of their affiliated organizations, or those of the publisher, the editors and the reviewers. Any product that may be evaluated in this article, or claim that may be made by its manufacturer, is not guaranteed or endorsed by the publisher.
